# Extraction of incarcerated medial epicondyle from the elbow joint using conventional nerve stimulator: a case report

**DOI:** 10.1186/1752-1947-8-329

**Published:** 2014-10-07

**Authors:** Sara Dorman, Sankur Sripada, Ben A Clift, Arpit Jariwala

**Affiliations:** 1 Department of Orthopaedic and Trauma, Royal Liverpool University Hospital, Liverpool L7 8XP, UK; 2 Department of Trauma and Orthopaedics, Ninewells Hospital and Medical School, Dundee DD1 9SY, UK

## Abstract

**Introduction:**

Incarceration of the medial epicondyle is a well-recognised sequelae following closed reduction of the elbow. Manipulation for extraction is not usually successful and hence an incarcerated medial epicondyle is usually an indication for open reduction and fixation.

**Case presentation:**

We describe a simple technique of closed reduction using a conventional nerve stimulator to extract an incarcerated medial epicondyle in a 13-year-old Caucasian boy. This technique uses contraction of the attached common flexor muscles to indirectly extract the trapped medial epicondyle.

**Conclusions:**

This is a simple technique using a commonly available nerve stimulator and may obviate the need for extensile open reduction for extraction of the incarcerated medial epicondyle. We would recommend this technique where closed reduction methods have failed.

## Introduction

Incarceration of the medial epicondyle (ME) is a well-recognised sequelae following closed reduction of the elbow [1,2]. Early diagnosis and prompt extraction of incarcerated ME is essential in preventing growth disturbance and disability [2,3]. Manipulation for extraction is not usually successful and hence an incarcerated ME is usually an indication for open reduction and fixation [1].

We describe a simple technique of closed reduction of an incarcerated ME using a conventional nerve stimulator. This technique uses contraction of the attached common flexor muscles to indirectly extract the trapped ME.

## Case presentation

**Figure 1:**
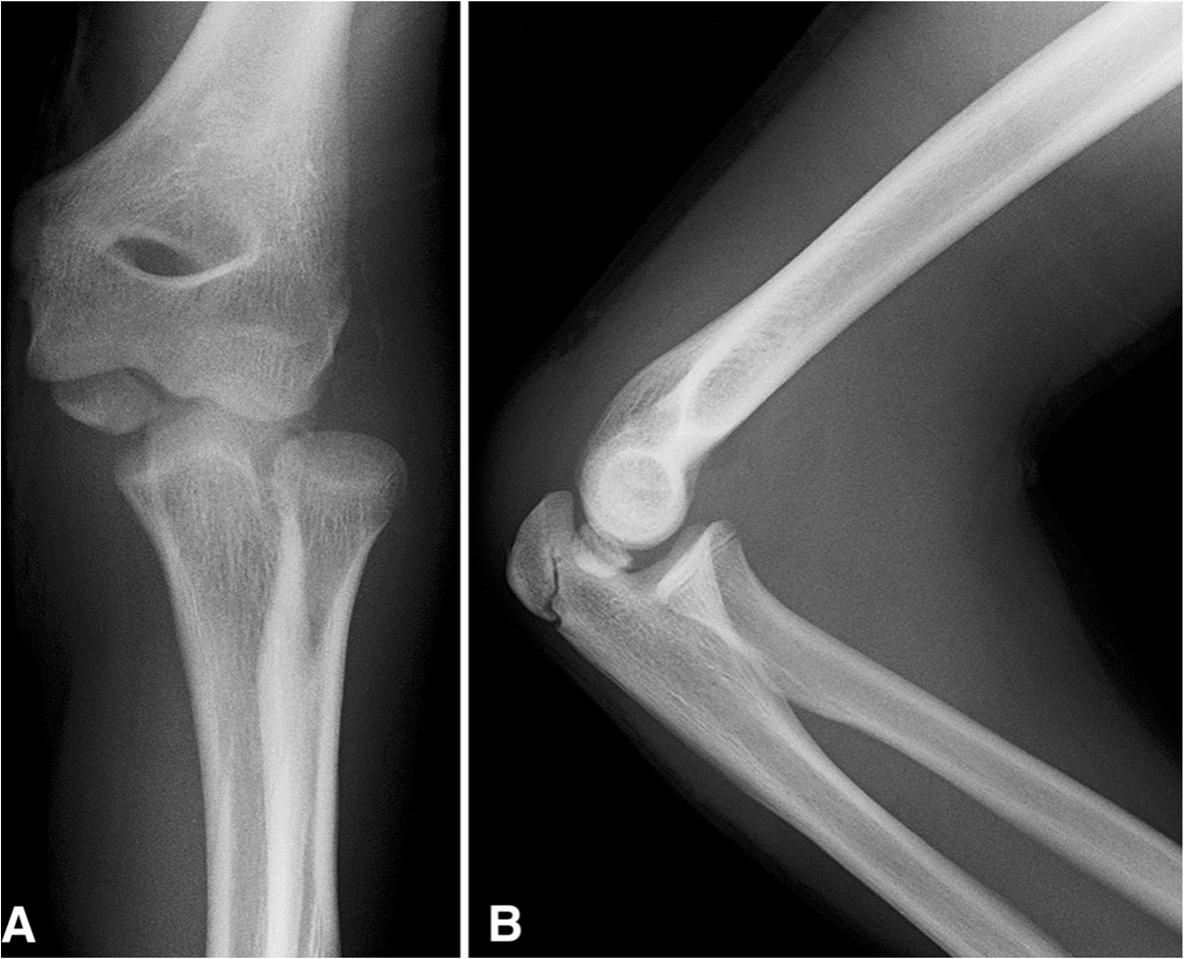
Anteroposterior radiograph (1A) and lateral radiograph (1B) of the right elbow demonstrating incarcerated medial epicondyle in the elbow joint following closed reduction.

**Figure 2:**
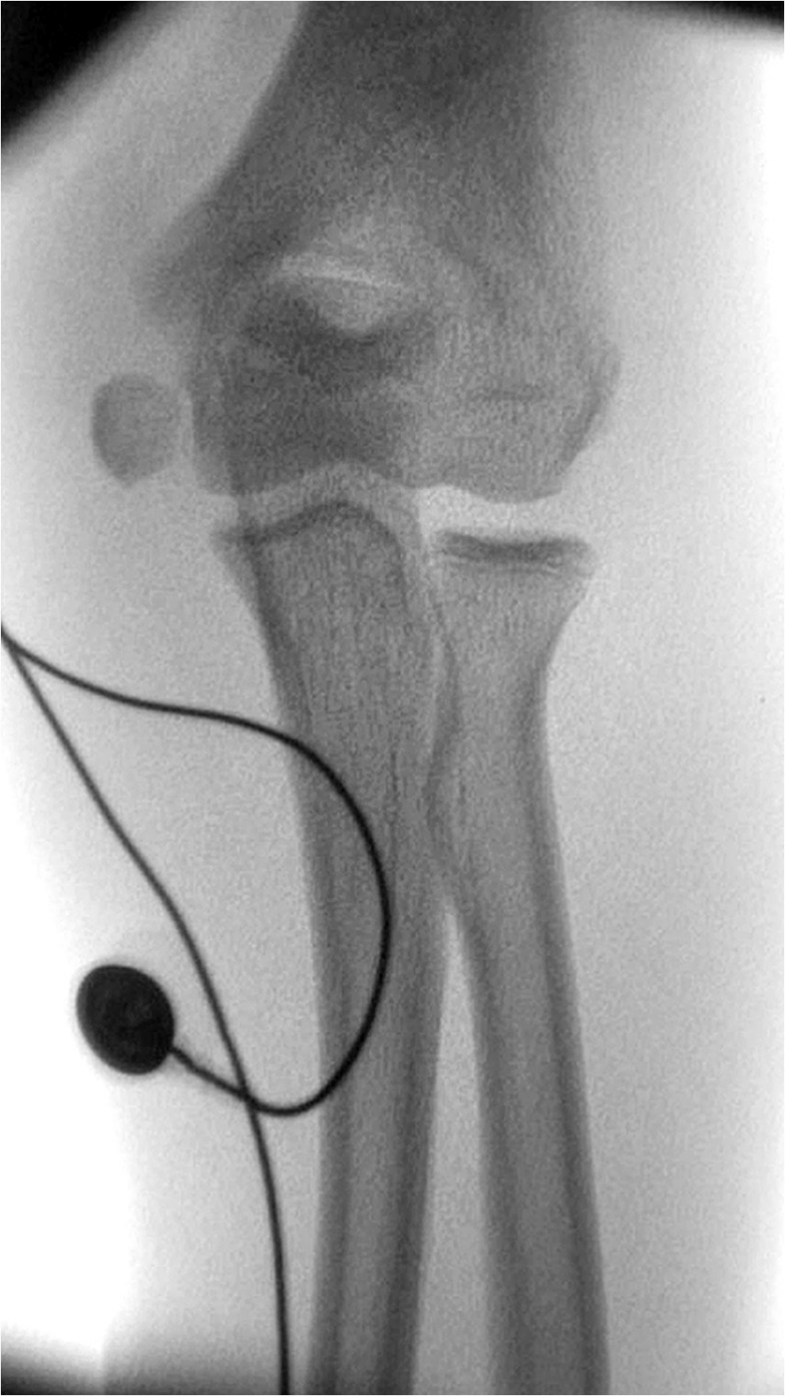
Image intensifier demonstrating medial epicondyle extraction with nerve stimulator.

A 13-year-old Caucasian boy presented with a left elbow dislocation to our Accident and Emergency department. Postreduction radiographs demonstrated an incarcerated ME in his joint and hence he was taken to theatre for further management (Figure 1A and 1B).Attempted closed extraction of the ME with valgus stress on his supinated forearm with dorsiflexion of his wrist failed. Hence the described technique was used with electrodes attached to the common flexor muscle mass on the medial aspect of his forearm. A summated and continuous stimulus was given using a commonly available nerve stimulator (Stimuplex® nerve stimulator, Braun). This resulted in a sudden significant contraction of his flexor group of muscles leading to the extraction of ME (Figure 2). After extraction the ME was still found to be displaced more than 5mm and therefore it was internally fixed using a cannulated screw.

On follow up he had no ulnar nerve symptoms, a good range of motion and the fracture had healed in an excellent position.

## Discussion

Humeral ME fractures account for up to 20% of all paediatric elbow fractures and 60% of these humeral ME fractures are associated with elbow dislocation [2]; ME incarceration is a well-recognised sequelae of elbow reduction [1].

Early diagnosis, anatomical reduction, and internal fixation are key to reducing the risk of growth disturbance, articular incongruence, and disability [3]. Traditionally, an incarcerated fragment is an absolute indication for open reduction. Relative indications include ulnar nerve dysfunction, high demand athletes and displacement >2mm [4].

Closed reduction of an incarcerated ME fragment using a conventional nerve stimulator uses contraction of the attached common flexor muscles to indirectly reduce the ME.

Due to the proximity of the ulnar nerve and potential fracture displacement it is probable that open reduction internal fixation may still be required. The general recommendation is that if after closed reduction of elbow or after extraction of the ME fragment through this approach more than 5mm displacement still persists, then it requires an internal fixation for optimal results.

This novel technique however facilitates a minimally invasive approach, reducing the amount of force applied, preventing complications such as soft tissue injury, fragment splitting and periosteal stripping caused by surgical instruments [5]. Furthermore this is the first known report of its kind and as such may have valid application for a wide range of avulsion fractures.

## Conclusions

This simple technique using a commonly available nerve stimulator may obviate the need for extensile open reduction [5] for extraction of an incarcerated ME. We would recommend this technique where other closed reduction methods have failed.

## Consent

Written informed consent was obtained from the patient’s legal guardian(s) for publication of this case report and any accompanying images. A copy of the written consent is available for review by the Editor-in-Chief of this journal.

## Competing interests

The authors declare that they have no competing interests.

## Authors’ contributions

AJ and SS were involved with the management of the case. SD reviewed the literature. SD, AJ, and SS were involved in writing the report. BC was the surgeon in charge of the case and helped in editing the report. All authors read and approved the final manuscript.
